# Comparing the performance of meta-classifiers—a case study on selected imbalanced data sets relevant for prediction of liver toxicity

**DOI:** 10.1007/s10822-018-0116-z

**Published:** 2018-04-06

**Authors:** Sankalp Jain, Eleni Kotsampasakou, Gerhard F. Ecker

**Affiliations:** 1 Department of Pharmaceutical Chemistry, University of Vienna, Althanstrasse 14, 1090 Vienna, Austria; 2 Present Address: Computational Toxicology Group, CMS, R&D Platform Technology & Science, GSK, Park Road, Ware, Hertfordshire SG12 0DP UK

**Keywords:** Imbalanced datasets, Machine learning, Classification model, Meta-classifiers, Stratified bagging, Cost sensitive classifier

## Abstract

**Abstract:**

Cheminformatics datasets used in classification problems, especially those related to biological or physicochemical properties, are often imbalanced. This presents a major challenge in development of in silico prediction models, as the traditional machine learning algorithms are known to work best on balanced datasets. The class imbalance introduces a bias in the performance of these algorithms due to their preference towards the majority class. Here, we present a comparison of the performance of seven different meta-classifiers for their ability to handle imbalanced datasets, whereby Random Forest is used as base-classifier. Four different datasets that are directly (cholestasis) or indirectly (via inhibition of organic anion transporting polypeptide 1B1 and 1B3) related to liver toxicity were chosen for this purpose. The imbalance ratio in these datasets ranges between 4:1 and 20:1 for negative and positive classes, respectively. Three different sets of molecular descriptors for model development were used, and their performance was assessed in 10-fold cross-validation and on an independent validation set. Stratified bagging, MetaCost and CostSensitiveClassifier were found to be the best performing among all the methods. While MetaCost and CostSensitiveClassifier provided better sensitivity values, Stratified Bagging resulted in high balanced accuracies.

**Graphical Abstract:**

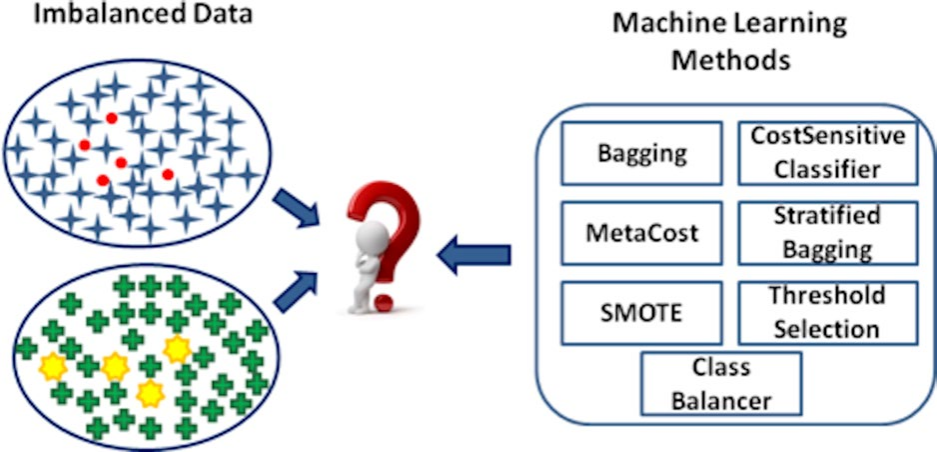

**Electronic supplementary material:**

The online version of this article (10.1007/s10822-018-0116-z) contains supplementary material, which is available to authorized users.

## Introduction

A wide range of classification and regression methods have been applied in QSAR studies. However, many classification methods assume that datasets are balanced in terms of the number of instances of each class and thus give equal importance to all classes, often resulting in classification models of poor accuracy [1, 2]. A major problem that arises in this context is class imbalance, i.e. the number of instances of one class substantially differ from those of the other classes. Especially in the field of drug discovery, imbalanced datasets [2–4] need to be frequently dealt with [2]. Characteristically, a classifier developed on an imbalanced data set shows a low error rate for the majority class and a high error rate for the minority class [5, 6]. Nevertheless, a few studies pointed out that the class imbalance is not a main obstacle in learning [7, 8], and several methods have been developed to address this issue. These methods can be broadly divided into (1) data-oriented/re-sampling techniques; (2) algorithm-oriented methods; and (3) combinatorial/ensemble/hybrid techniques [2, 3, 7, 9, 10].

Several studies compared classifiers that handle imbalanced datasets. Schierz et al. [11] compared four WEKA classifiers (Naïve Bayes, SVM, Random Forest and J48 tree) and reported SVM and J48 to be the best performing for bioassay datasets. Lin and Chen in 2013 found SVM threshold adjustment as the best performing classifier (among linear discriminant analysis, Random Forest, SVM and SVM-threshold adjustment) to deal with imbalanced HTS datasets [9]. Later, Zakarov et al. used under-sampling and threshold selection techniques on several imbalanced PubChem HTS assays to test and develop robust QSAR models in the program GUSAR [12]. In a recent study, Razzaghi et al. reported multilevel SVM-based algorithms to outperform conventional SVM, weighted SVM, neural networks, linear regression, Naïve Bayes and C4.5 tree using public benchmark datasets having imbalanced classes and missing values and real data in health applications [13].

A comprehensive comparison of the performance of different meta-classifiers on datasets with different levels of class imbalance, which would provide guidance for choosing the appropriate method for an imbalanced dataset, has not been attempted so far. Herein, we evaluated the performance of seven distinct meta-classifiers from the three aforementioned categories on four datasets from the toxicology domain. The imbalance ratio of the datasets ranges from 1:4 to 1:20 for the positive and the negative class, respectively. The meta-classifiers were applied to build classification models based on three different sets of descriptors. Considering its wide applicability in modeling imbalanced datasets, Random Forest was used as the common base-classifier for all models [14–18]. Further, we discuss the reasons behind the superior performance of certain meta-classifiers in comparison to the others while explaining their intrinsic limitations.

## Methods

### Training datasets

Four different datasets from the biomedical sciences domain were used in this study. Two of these are the OATP1B1 and OATP1B3 inhibition datasets consisting of 1708 and 1725 compounds, respectively. Both were compiled and used in our previous study that reported classification models for OATP1B1 and 1B3 inhibition [19]. The other two datasets come from the toxicology domain and are related to drug-induced cholestasis for human data and animal data which comprise 1766 and 1578 compounds, respectively. Both datasets were published in a previous study that reported computational models for hepatotoxicity and other liver toxicity endpoints [20].

### External test datasets

The external test sets for OATP1B1 and 1B3 inhibition from our previous study served as test datasets in this study [19]. The test set for human cholestasis was compiled in two stages from two previous studies [21]. The positives for human cholestasis were compiled from literature [22–25] and from the SIDER v2 database [26, 27]. As cholestasis is one of the three types of drug induced liver injury (DILI), and the compounds that are negative for DILI will also be negative for cholestasis, the negatives for drug-induced liver injury compiled in a previous study [21] were used as negatives for cholestasis. Overall, the external human cholestasis dataset consisted of 231 compounds. No data were available for animal cholestasis to be used as an external test dataset. The composition and degree of class imbalance of each training and test dataset is presented in Table 1.

**Table 1:** An overview of the training and test datasets used in this study

Dataset name	Total number of compounds	Number of positives	Number of negatives	Imbalance ratio (negatives: positives)	Source
OATP1B1 inhibition training	1708	190	1518	8:1	Kotsampasakou et al. [19]
OATP1B1 inhibition testing	201	64	137	2:1	Kotsampasakou et al. [19]
OATP1B3 inhibition training	1725	124	1601	13:1	Kotsampasakou et al. [19]
OATP1B3 inhibition testing	209	40	169	4:1	Kotsampasakou et al. [19]
Cholestasis human training	1766	347	1419	4:1	Mulliner et al. [20]
Cholestasis human testing	231	53	178	3:1	Kotsampasakou et al. [21]
Cholestasis animal training	1578	75	1503	20:1	Mulliner et al. [20]

The chemotypes in the datasets were curated using the following protocol:

- Removed all inorganic compounds according to chemical formula in MOE 2014.09 [28].
- Removed salts and compounds containing metals and/or rare or special atoms.
- Standardized chemical structures using Francis Atkinson Standardiser tool [29].
- Removed duplicates and permanently charged compounds using MOE 2014.09 [28].
- 3D structures were then generated using CORINA (version 3.4) [30], and energy minimized with MOE 2014.09 [28], using default settings (Forcefield MMF94x, gradient 0.05 RMS kcal/mol/A^2^, preserving chirality).

### Molecular descriptors

Three different sets of descriptors were calculated for each of the datasets:

1. All 2D MOE [28] descriptors (192 descriptors in total).
2. ECFP6 fingerprints (1024 bits) calculated with RDKit [31].
3. MACCS fingerprints (166 bits), calculated with PaDEL software [32].

## Machine learning methods

Random Forest [33] implemented in the WEKA software suite [34, 35] was used as a base-classifier along with all the meta-learning methods evaluated in this study. The number of trees was arbitrarily set to 100 (default), since it has been shown that the optimal number of trees is usually 64–128, while further increasing the number of trees does not necessarily improve the model’s performance [36]. The following meta-classifiers were investigated: (1) Bagging, (2) Under-sampled stratified bagging, (3) Cost-sensitive classifier, (4) MetaCost, (5) Threshold Selection, (6) SMOTE and (7) ClassBalancer.

1. *Bagging* (*Bootstrap AGGregatING*) [37] is a machine learning technique that is based on an ensemble of models developed using multiple training datasets sampled from the original training set. It calculates several models and averages them to produce a final ensemble model [37]. A traditional bagging method generates multiple copies of the training set by selecting the molecules with replacement from training set in a random fashion. Because of random sampling, about 37% of the molecules are not selected and left out in each run. These samples create the “out-of-the-bag” sets, which are used for testing the performance of the final model. A total of 64 models were used for our analysis, since it was shown in an earlier study by Tetko et al. [38] that larger numbers of models per ensemble (e.g. 128, 256, 512 and 1024) did not significantly increase the balanced accuracy of models.
2. *Under-sampled stratified bagging* [2, 8, 38] In this method, the total bagging training set size is double the number of the minority class molecules. Although a small set of samples was selected each time, the majority of molecules contributed to the overall bagging procedure, since the datasets were generated randomly. The performance of the developed models is tested with molecules from the “out-of-the-bag” set [38]. Since only one way of stratified learning, i.e., under-sampling stratified bagging, was used in the study, we refer to it as “Stratified Bagging”.
  Bagging and Stratified Bagging were used as implemented in the Online Chemical Modeling Environment (OCHEM) [39, 40]. For other meta-classifiers, WEKA(v. 3-7-12) [34, 35] was used.
3. *Cost sensitive classifier* [2–4, 10, 11] is a meta-classifier that renders the base classifier cost-sensitive. Two methods can be used to introduce cost-sensitivity: (i) reweighting training instances according to the total cost assigned to each class, i.e. the weights are applied during learning, or; (ii) predicting the class with minimum expected misclassification cost (rather than the most likely class), i.e. the “cost-sensitive” is introduced in the test phase. In our case, the cost sensitivity was introduced according to method (i) using the CostSensitiveClassifier from the set of meta-classifiers of the WEKA software [34, 35].
4. *MetaCost* [41] is another application that provides the methodology to perform cost-sensitive training of a classifier in a generalized meta-learning manner independent of the underlying classifier. It is a combination of Cost-sensitive meta-classifier and Bagging [37]. The algorithm uses class-relabeling, i.e. it modifies the original training set by changing the class labels to the so-called “optimal classes”. The classifier is then trained on this modified training set, which results in having the error rate minimized according to the cost matrix provided to the MetaCost algorithm. This implementation uses all bagging iterations when reclassifying training data. MetaCost is advantageous as, unlike CostSensitiveClassifier, a single cost-sensitive classifier of the base learner is generated, thus giving the benefits of fast classification and interpretable output (if the base learner itself is interpretable). MetaCost further differs from traditional bagging by the fact that the number of examples in each resample may be smaller than the training set size. This variation improves the efficiency of the algorithm. More details about the method can be found in [41].
  For both CostSensitiveClassifier and MetaCost, several trials of different cost matrices were applied, until a satisfactory outcome was retrieved.
5. *ThresholdSelector* [42] is a meta-classifier implemented in WEKA [34, 35] that sets a threshold on the probability output of a base-classifier. Threshold adjustment for the classifier’s decision is one of the methods used for dealing with imbalanced datasets [2, 43]. By default, the WEKA probability threshold to assign a class is 0.5, i.e. if an instance is attributed with a probability of equal or less than 0.5, it is classified as negative for the respective class, while if it is greater than 0.5, the instance is classified as positive. For our study, the optimal threshold was selected automatically by the meta-classifier by applying internal fivefold cross validation to optimize the threshold according to FMeasure (Eq. 7), a measure of a model’s accuracy which considers both precision and sensitivity [44].
6. *SMOTE* [45] (*Synthetic minority over-sampling technique*) increases the minority class by generating new “synthetic” instances based on its number of nearest neighbours. SMOTE, as implemented in WEKA, was used to generate synthetic examples. For our study, five nearest neighbours of a real existing instance (minority class) were used to compute a new synthetic one. For different datasets, different percentages of SMOTE instances were created, which can be found in the supplementary information (Table S1). The complete algorithm is explained in [45].
7. *ClassBalancer* [34, 35, 46] reweights the instances so that the sum of weights for all classes of instances in the data is the same, i.e. the total sum of weights across all instances is maintained. This is an additional way to treat class imbalance, unlike CostSensitiveClassifier or MetaCost, which try to minimize the total misclassification cost.

With respect to parameters, not for all classifiers a parameter optimization was performed. For instance, no parameters were adjusted for ClassBalancer since it automatically reassigns weights to the instances in the dataset such that each class has the same total weight [46]. For Bagging and Stratified Bagging, the only parameter to optimize would be the number of bags. In our case, the number of bags was adjusted to 64 as a previous study [38] suggests that generation of 64 models provides satisfactory results without exponentially increasing the computational cost. In case of ThresholdSelector, an optimal threshold was selected automatically via fivefold cross-validation before selecting the final model on the basis of FMeasure. For both CostSensitiveClassifier and MetaCost, the cost for misclassification was initially applied in accordance with the imbalance ratio, which, in case it did not provide a sensitivity of at least 0.5, was further increased to arrive at the final model. In case of SMOTE, similar principles were applied: initially, the number of the synthetic instances created was set to a number that balances the two classes. If insufficient, it was further increased until no further improvement in sensitivity (with no reduction in specificity) was observed. The detailed parameter settings of the best performing models for each method are provided in the supplementary material (Table S1).

### Validation

All models were evaluated in a 10-fold cross-validation followed by an external validation performed on independent test sets, except for Bagging and Stratified Bagging. For Bagging and Stratified Bagging, since multiple training datasets were generated by selecting the molecules with replacement from training set in a random fashion, this leaves out about 37% of the instances in each run. Therefore, these molecules that constitute the ‘out-of-the-bag’ sets are later used for testing the performance of the final model.

## Model performance assessment: selection of the optimal method

Prior to identifying the best performing method, an optimal model for each meta-classifier was selected. The best parameters for the model were selected using linear search (as explained in the “Methods” section). For all models, different performance measures including sensitivity (Eq. 1), specificity (Eq. 2), accuracy (Eq. 3), balanced accuracy (Eq. 4), Matthews correlation coefficient (MCC, Eq. 5), area under the curve (AUC) and precision (Eq. 6) were calculated. A model was considered eligible for selection if the 10-fold cross-validation provided a sensitivity value of at least 0.5 and a specificity value not less than 0.5. As the datasets are relevant to different toxicological endpoints, sensitivity was considered more important. For a highly imbalanced dataset, accuracy may be misleading. Therefore we considered balanced accuracy (which considers both sensitivity and specificity) as a more appropriate performance measure to compare different classifiers for their ability to handle imbalanced datasets. If two models provided the same sensitivity, the model that demonstrated higher balanced accuracy was prioritized for selection. Furthermore, 20 iterations were performed by varying the seed for cross validation [by assigning values from 1 (default) to 20]. For Bagging and Stratified Bagging, the 20 iterations were performed by changing the random seed for the Random Forest generation by assigning values from 1 (default) to 20. After cross-validation, average values for different performance measures were calculated and compared. The best method was then evaluated by performing a statistical t-test in R [47], as well as on the basis of the performance on external test sets. The individual settings used in selecting the best model for each meta-classifier can be found in the supplementary information (Table S1).

1 $$Sensitivity=~\frac{{TP}}{{(TP+FN)}}$$

2 $$Specificity=~\frac{{TN}}{{(TN+FP)}}$$

3 $$Accuracy=\frac{{(TP+TN)}}{{(TP+FP+TN+FN)}}$$

4 $$Balanced~Accuracy=\frac{1}{2}\left( {\frac{{(TP)}}{{(TP+NP)}}+\left. {\frac{{(TN)}}{{(TN+FP)}}} \right)} \right.$$

5 $$MCC = \frac{{\{ \left( {TP \times TN} \right) - (FP \times FN)\} }}{{\{ \left( {TP + FP} \right) \times \left( {TP + FN} \right) \times \left( {TN + FP} \right) \times (TN + FN)\} ^{{1/2}} }}$$

6 $$Precision=\frac{{(TP)}}{{(TP+FP)}}$$

7 $$FMeasure=\frac{{2TP}}{{(2TP+FP+FN)}}$$

TP: true positives; TN: true negatives; FP: false positives; FN: false negatives.

## Results and discussion

**Fig. 1:**
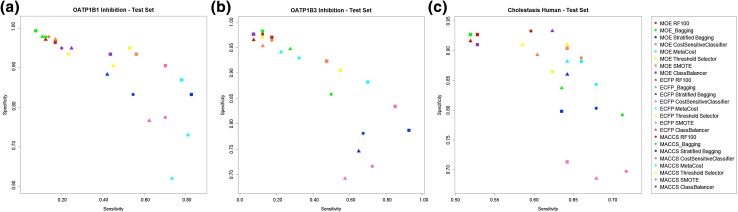
Comparison of performances of different meta-classifiers on test sets **a** OATP1B1 inhibition **b** OATP1B3 inhibition **c** human cholestasis. *x-axis* corresponds to the sensitivity and on the *y-axis* is the specificity. The squares correspond to MOE descriptors, the triangles correspond to ECFP6 fingerprints and the circles correspond to MACCS fingerprints. Each classifier is depicted in a different color: red for RF standalone, green for Bagging, blue for Stratified Bagging, dark pink for CostSensitiveClassifier, cyan for MetaCost, yellow for ThresholdSelector, orange for SMOTE and dark violet for ClassBalancer. Please note that the scaling for the two axes are different

Tables S2–S5 in the supplementary material report the performance measures for predictions on all datasets used in this study. The performance values of the base-classifier (Random Forest) are also reported to facilitate a comparison with the investigated methods. For each dataset, the mean and the standard deviation values of performance of the best performing models (based on 20 iterations) were calculated and are reported in Tables S6–S9 (supplementary material). Figure 1a–c, Figure S1(a–d) in the supplementary material provide a comparison of performances of different meta-classifiers on the three test datasets (no test set available for animal cholestasis) and four training sets respectively.

Irrespective of the dataset and the descriptor set used, Random Forest was found to be the weakest performing classifier as anticipated. Except on the test dataset for human cholestasis, Random Forest alone did not yield a sensitivity greater than 0.5, which indicates that assistance of a meta-classifier indeed consistently improves performance when handling imbalanced datasets. Among the Meta-Classifier based methods, bagging provided the lowest performance. A simple reason behind the failure of Bagging is that it only does resampling without any effort to balance or weight the two classes.

Threshold Selection was frequently found to be among the good performing methods. In many cases, this classifier could handle imbalance very well. However, the sensitivity measures were poor in comparison to other classifiers. This could be due to the fact that the thresholds were selected on the basis of FMeasure, as accuracy and specificity are not suitable due to the high impact of the majority class. If the selection of best models is done purely on the basis of sensitivity, this classifier yields very good sensitivity values (0.8–1.0), however with a radical decrease in specificity (0.2–0). Notably, Threshold Selection provided better results in combination with a second meta-classifier. But since the aim of the study was to compare the classifiers individually, this trend was not investigated further.

Stratified Bagging, CostSensitiveClassifier and MetaCost were consistently the best performing classifiers in both cross-validation and test set validation for all the datasets (see Fig. 1, Figure S1 in the supplementary material). Further, the t-test on the basis of 95% confidence interval (exact p-values not shown here) indicated a statistically significant difference in performance between the selected methods (meta-classifiers). The statistical test was performed pair-wise for all the obtained performance measures, with more stress on sensitivity and balanced accuracy. Both MetaCost and CostSensitiveClassifier tended to yield higher sensitivities while Stratified Bagging, on the other hand, was found to be superior in terms of MCC, balanced accuracy and AUC. An advantage of Stratified Bagging is that it is a straightforward method with only one parameter to optimize, i.e. the number of bags. On the other hand, cost-sensitive approaches tend to give more weight to sensitivity when needed, which is an advantage for toxicity prediction. Although both methods provided comparable performances, the cost that had to be applied was greater in case of CostSensitiveClassifier in comparison to MetaCost. This is due to the fact that the latter is a hybrid classifier which combines Bagging with the application of a cost, thus equilibrating the dataset more easily. It should further be noted that the computational cost for MetaCost is higher than that for CostSensitiveClassifier. On the other hand, Stratified Bagging is not computationally demanding (for the optimal parameter of 64 bags). Since each bag is double the size of the minority class, the calculation of models using Stratified Bagging requires less computational time, compared to the models built using Bagging (the bags are of the same size as the training set) and MetaCost (includes both bagging and weighting).

SMOTE and ClassBalancer were only in a few cases able to provide a sensitivity of at least 0.5 in both cross-validation and test set evaluation. Considering its reputation in handling such problems, the poor performance of SMOTE was quite surprising. We assume that the small size of the datasets could be the primary reason behind SMOTE’s poor performance. The datasets used in this study are much smaller in size compared to the HTS datasets in which the minority class has enough instances for SMOTE to generate synthetic instances, although the overall imbalance ratio is typically in the range of 100:1 [12, 45, 48].

With respect to the different sets of descriptors used, the performance of the classifiers on different datasets remained almost the same. Of all the descriptors, 2D MOE descriptors and MACCS fingerprints provided the best performance across many of the datasets, while ECFP6 fingerprints consistently performed lower. Considering the amount of information encoded in ECFP6 (1024 bits) in comparison to MACCS fingerprints (166 bits) and the MOE descriptors, it might be assumed that the poor performance of ECFP6 is subject to the individual datasets in this study. This also highlights the fact that sometimes simple set of descriptors could provide better results than complex and highly populated descriptors. Moreover, in other recent studies [49–51] different descriptor and fingerprint combinations did not demonstrate significant differences in performance.

Overall, the best classifiers performed well regardless of the type of data (toxicity endpoint or a general or specific in vitro endpoint), the type and number of descriptor sets used, or the degree of class imbalance. However, there were instances where a dataset related to in vivo toxicity (animal cholestasis) could not be successfully handled by the best classifiers. Finally, highly sophisticated meta-classifiers such as Stratified Bagging and MetaCost, that combine re-sampling and a way to weight the two classes, performed in principle better than Bagging and ClassBalancer.

## Conclusions

In this study, we compared the performance of seven different meta-classifiers for their ability to handle imbalanced datasets. We demonstrated that, for all datasets used in the study, Stratified Bagging performed at least as good as cost-sensitive approaches such as MetaCost and CostSensitiveClassifier and in most cases outperformed them. Random Forest (as a standalone classifier) and Bagging were unable to address the imbalance issue. Interestingly, the choice of descriptors did not play a substantial role in ranking the performance of different classifiers. Thus, considering that Stratified Bagging can be directly used in combination with any machine-learning method without parameter optimization, a general recommendation for handling imbalanced datasets is to wrap the modeling process in the stratified bagging loop. However, one should also consider the computational cost, as extensive re-sampling can be computationally expensive. Therefore, a method that balances between the complexity of the algorithm and computational cost would be an ideal choice to obtain optimal results.

## Electronic supplementary material

Below is the link to the electronic supplementary material.

Supplementary material 1 (DOCX 167 KB)

## Abbreviations

AUC: Area under the ROC curve
HTS: High throughput screening
MCC: Matthews correlation coefficient
OATP1B1: Organic anion transporting polypeptide 1B1
OATP1B3: Anion transporting polypeptide 1B3
RF: Random Forest
sd: Standard deviation
SMOTE: Synthetic minority over-sampling technique
SVM: Support vector machines
