# Supplementary material for: Comparing the performance of meta-classifiers—a case study on selected imbalanced data sets relevant for prediction of liver toxicity
Source: J Comput Aided Mol Des. 2018 Apr 6;32(5):583–90. doi: 10.1007/s10822-018-0116-z (PMC5919997; doi:10.1007/s10822-018-0116-z)

**Comparing the performance of meta-classifiers – A case study on selected imbalanced data sets relevant for prediction of liver toxicity**

Sankalp Jain^a,§^, Eleni Kotsampasakou^a,§,#^ and Gerhard F. Ecker^a,*^

*^a^University of Vienna, Department of Pharmaceutical Chemistry, Althanstrasse 14, 1090 Vienna, Austria*

*^#^present address: Computational Toxicology Group, CMS, R&D Platform Technology & Science, GSK, Park Road, Ware, Hertfordshire, SG12 0DP, United Kingdom*

*^§^both authors contributed equally to this manuscript*

*Corresponding author: E-Mail: [gerhard.f.ecker@univie.ac.at](mailto:gerhard.f.ecker@univie.ac.at); Phone: +43-1-4277-55110; eFax: +43-1-4277-855110

**Supplementary material**

**Tables**

**Table S1.**Tuned settings of the best performing models for each meta-classifier/method

**a.** OATP1B1 dataset

| **Method** | **2D MOE descriptors** | **ECFP6 fingerprints** | **MACCS fingerprints** |
| --- | --- | --- | --- |
| **Stratified Bagging** | - | - | - |
| **CostSensitiveClassifier** | cost 30:1  matrix: [0.0, 1.0; 30.0, 0.0] | cost 100:1  matrix: [0.0, 1.0; 100.0, 0.0] | cost 100:1  matrix: [0.0, 1.0; 100.0, 0.0] |
| **MetaCost** | cost 10:1  matrix: [0.0, 1.0; 10.0, 0.0] | cost 30:1  matrix: [0.0, 1.0; 30.0, 0.0] | cost 25:1  matrix: [0.0, 1.0; 25.0, 0.0] |
| **SMOTE** | 1500% synthetic instances | 2000% synthetic instances | 1500% synthetic instances |

**b.** OATP1B3 dataset

| **Method** | **2D MOE descriptors** | **ECFP6 fingerprints** | **MACCS fingerprints** |
| --- | --- | --- | --- |
| **Stratified Bagging** | - | - | - |
| **CostSensitiveClassifier** | cost 70:1  matrix: [0.0, 1.0; 70.0, 0.0] | cost 280:1  matrix: [0.0, 1.0; 280.0, 0.0] | cost 200:1  matrix: [0.0, 1.0; 200.0, 0.0] |
| **MetaCost** | cost 13:1  matrix: [0.0, 1.0; 13.0, 0.0] | cost 50:1  matrix: [0.0, 1.0; 50.0, 0.0] | cost 40:1  matrix: [0.0, 1.0; 40.0, 0.0] |
| **SMOTE** | 1500% synthetic instances | 2000% synthetic instances | 1300% synthetic instances |

**c.** Cholestasis human dataset

| **Method** | **2D MOE descriptors** | **ECFP6 fingerprints** | **MACCS fingerprints** |
| --- | --- | --- | --- |
| **Stratified Bagging** | cost 2:1 | cost 2:1 | cost 2:1 |
| **CostSensitiveClassifier** | cost 14:1  matrix: [0.0, 1.0; 14.0, 0.0] | cost 12:1  matrix: [0.0, 1.0; 12.0, 0.0] | cost 12:1  matrix: [0.0, 1.0; 12.0, 0.0] |
| **MetaCost** | cost 8:1  matrix: [0.0, 1.0; 8.0, 0.0] | cost 8:1  matrix: [0.0, 1.0; 8.0, 0.0] | cost 8:1  matrix: [0.0, 1.0; 8.0, 0.0] |
| **SMOTE** | 1300% synthetic instances | 3000% synthetic instances | 1300% synthetic instances |

cost 2:1 for Stratified Classifier: Stratified bagging used in combination with MetaCost with matrix: [0.0, 1.0; 2.0, 0.0]. For the case of human cholestasis dataset, Stratified Bagging on its own was not able to handle the dataset in such satisfactory way.  Thus Stratified Bagging was combined with the application of a slight cost of 2:1 in favor of the minority class

**d.** Cholestasis animal dataset

| **Method** | **2D MOE descriptors** | **ECFP6 fingerprints** | **MACCS fingerprints** |
| --- | --- | --- | --- |
| **Stratified Bagging** | cost 2:1 | cost 2:1 | cost 2:1 |
| **CostSensitiveClassifier** | cost 450:1  matrix: [0.0, 1.0; 450.0, 0.0] | cost 500:1  matrix: [0.0, 1.0; 500.0, 0.0] | cost 500:1  matrix: [0.0, 1.0; 500.0, 0.0] |
| **MetaCost** | cost 45:1  matrix: [0.0, 1.0; 45.0, 0.0] | cost 45:1  matrix: [0.0, 1.0; 45.0, 0.0] | cost 50:1  matrix: [0.0, 1.0; 50.0, 0.0] |
| **SMOTE** | 3000% synthetic instances | 3000% synthetic instances | 3000% synthetic instances |

cost 2:1 for Stratified Classifier: Stratified bagging used in combination with MetaCost with matrix: [0.0, 1.0; 2.0, 0.0]. For the case of animal cholestasis dataset, Stratified Bagging on its own was not able to handle the dataset in such satisfactory way.  Thus Stratified Bagging was combined with the application of a slight cost of 2:1 in favor of the minority class.

**Table S2.** Results on OATP1B1 inhibition dataset for all calculated statistics metrics : Accuracy, Balanced Accuracy, Sensitivity, Specificity, MCC, AUC, Precision. The performance is given for both 10-fold cross-validation and on the external test set. With bold font are depicted those models that gave a satisfactory result of sensitivity > 0.5 and they were further investigated by performing 20 iterations.

| Model Settings | Descriptors | Validation | Accuracy | Balanced Accuracy | Sensitivity | Specificity | MCC | AUC | Precision |
| --- | --- | --- | --- | --- | --- | --- | --- | --- | --- |
| Random Forest | MOE | 10 CV | 0.893 | 0.614 | 0.253 | 0.974 | 0.322 | 0.809 | 0.545 |
|  |  | Test set | 0.711 | 0.568 | 0.172 | 0.964 | 0.233 | 0.837 | 0.688 |
|  | ECFP6 | 10 CV | 0.892 | 0.573 | 0.163 | 0.983 | 0.256 | 0.798 | 0.544 |
|  |  | Test set | 0.701 | 0.548 | 0.125 | 0.971 | 0.188 | 0.804 | 0.667 |
|  | MACCS | 10 CV | 0.899 | 0.623 | 0.268 | 0.978 | 0.356 | 0.778 | 0.6 |
|  |  | Test set | 0.711 | 0.560 | 0.141 | 0.978 | 0.233 | 0.768 | 0.75 |
| Bagging | MOE | Training set | 0.897 | 0.616 | 0.254 | 0.978 | 0.340 | 0.74 | 0.585 |
|  |  | Test set | 0.701 | 0.535 | 0.078 | 0.993 | 0.194 | 0.724 | 0.833 |
|  | ECFP6 | Training set | 0.892 | 0.585 | 0.190 | 0.979 | 0.272 | 0.694 | 0.529 |
|  |  | Test set | 0.701 | 0.544 | 0.109 | 0.978 | 0.211 | 0.572 | 0.700 |
|  | MACCS | Training set | 0.904 | 0.636 | 0.291 | 0.981 | 0.394 | 0.701 | 0.655 |
|  |  | Test set | 0.706 | 0.552 | 0.125 | 0.978 | 0.187 | 0.572 | 0.727 |
| Stratified Bagging | **MOE** | **Training set** | **0.809** | **0.768** | **0.714** | **0.821** | **0.395** | **0.819** | **0.333** |
|  |  | **Test set** | **0.831** | **0.830** | **0.828** | **0.832** | **0.634** | **0.887** | **0.697** |
|  | ECFP6 | **Training set** | **0.807** | **0.736** | **0.646** | **0.827** | **0.354** | **0.790** | **0.317** |
|  |  | Test set | 0.736 | 0.653 | 0.422 | 0.883 | 0.347 | 0.774 | 0.628 |
|  | **MACCS** | **Training set** | **0.783** | **0.757** | **0.725** | **0.790** | **0.365** | **0.798** | **0.300** |
|  |  | **Test set** | **0.741** | **0.689** | **0.547** | **0.832** | **0.390** | **0.809** | **0.603** |
| CostSensitive  Classifier | **MOE** | **10 CV** | **0.843** | **0.719** | **0.621** | **0.817** | **0.399** | **0.822** | **0.376** |
|  |  | **Test set** | **0.841** | **0.804** | **0.703** | **0.905** | **0.625** | **0.856** | **0.776** |
|  | **ECFP6** | **10 CV** | **0.653** | **0.711** | **0.784** | **0.637** | **0.269** | **0.791** | **0.213** |
|  |  | **Test set** | **0.721** | **0.670** | **0.625** | **0.766** | **0.38** | **0.789** | **0.556** |
|  | **MACCS** | **10 CV** | **0.645** | **0.701** | **0.774** | **0.628** | **0.257** | **0.79** | **0.207** |
|  |  | **Test set** | **0.751** | **0.739** | **0.703** | **0.774** | **0.458** | **0.779** | **0.592** |
| MetaCost | **MOE** | **10 CV** | **0.819** | **0.746** | **0.653** | **0.839** | **0.376** | **0.826** | **0.337** |
|  |  | **Test set** | **0.841** | **0.825** | **0.781** | **0.869** | **0.64** | **0.87** | **0.735** |
|  | **ECFP6** | **10 CV** | **0.622** | **0.693** | **0.784** | **0.602** | **0.245** | **0.769** | **0.198** |
|  |  | **Test set** | **0.657** | **0.677** | **0.734** | **0.62** | **0.331** | **0.758** | **0.475** |
|  | **MACCS** | **10 CV** | **0.673** | **0.703** | **0.742** | **0.664** | **0.263** | **0.767** | **0.217** |
|  |  | **Test set** | **0.756** | **0.772** | **0.813** | **0.73** | **0.509** | **0.769** | **0.584** |
| Threshold  Selector | **MOE** | **10 CV** | **0.881** | **0.721** | **0.516** | **0.926** | **0.423** | **0.806** | **0.467** |
|  |  | **Test set** | **0.816** | **0.740** | **0.531** | **0.949** | **0.555** | **0.837** | **0.829** |
|  | ECFP6 | **10 CV** | **0.868** | **0.712** | **0.511** | **0.912** | **0.390** | **0.797** | **0.422** |
|  |  | Test set | 0.761 | 0.679 | 0.453 | 0.905 | 0.41 | 0.804 | 0.69 |
|  | MACCS | 10 CV | 0.880 | 0.656 | 0.368 | 0.944 | 0.342 | 0.775 | 0.452 |
|  |  | Test set | 0.711 | 0.584 | 0.234 | 0.934 | 0.242 | 0.768 | 0.625 |
| SMOTE | **MOE** | **10 CV** | **0.869** | **0.710** | **0.505** | **0.914** | **0.389** | **0.807** | **0.425** |
|  |  | **Test set** | **0.816** | **0.749** | **0.563** | **0.934** | **0.555** | **0.823** | **0.800** |
|  | ECFP6 | 10 CV | 0.896 | 0.620 | 0.263 | 0.976 | 0.341 | 0.791 | 0.575 |
|  |  | Test set | 0.716 | 0.572 | 0.172 | 0.971 | 0.253 | 0.767 | 0.733 |
|  | MACCS | 10 CV | 0.898 | 0.657 | 0.347 | 0.966 | 0.391 | 0.777 | 0.564 |
|  |  | Test set | 0.711 | 0.560 | 0.141 | 0.978 | 0.233 | 0.787 | 0.75 |
| ClassBalancer | **MOE** | **10 CV** | **0.694** | **0.748** | **0.816** | **0.679** | **0.321** | **0.823** | **0.241** |
|  |  | Test set | 0.776 | 0.686 | 0.438 | 0.934 | 0.447 | 0.839 | 0.757 |
|  | ECFP6 | 10 CV | 0.893 | 0.657 | 0.353 | 0.961 | 0.377 | 0.799 | 0.532 |
|  |  | Test set | 0.726 | 0.6 | 0.25 | 0.949 | 0.291 | 0.805 | 0.696 |
|  | MACCS | 10 CV | 0.875 | 0.665 | 0.395 | 0.935 | 0.344 | 0.774 | 0.434 |
|  |  | Test set | 0.711 | 0.56 | 0.203 | 0.949 | 0.237 | 0.786 | 0.65 |

**Table S3.** Results on OATP1B3 inhibition dataset for all calculated statistics metrics : Accuracy, Balanced Accuracy, Sensitivity, Specificity, MCC, AUC, Precision. The performance is given for both 10-fold cross-validation and on the external test set. With bold font are depicted those models that gave a satisfactory result of sensitivity > 0.5 and they were further investigated by performing 20 iterations.

| Model Settings | Descriptors | Validation | Accuracy | Balanced Accuracy | Sensitivity | Specificity | MCC | AUC | Precision |
| --- | --- | --- | --- | --- | --- | --- | --- | --- | --- |
| Random Forest | MOE | 10 CV | 0.926 | 0.593 | 0.202 | 0.983 | 0.276 | 0.868 | 0.472 |
|  |  | Test set | 0.818 | 0.573 | 0.175 | 0.97 | 0.246 | 0.912 | 0.583 |
|  | ECFP6 | 10 CV | 0.926 | 0.540 | 0.089 | 0.991 | 0.168 | 0.841 | 0.423 |
|  |  | Test set | 0.804 | 0.526 | 0.075 | 0.976 | 0.112 | 0.795 | 0.429 |
|  | MACCS | 10 CV | 0.926 | 0.596 | 0.210 | 0.981 | 0.278 | 0.813 | 0.464 |
|  |  | Test set | 0.804 | 0.526 | 0.075 | 0.976 | 0.112 | 0.821 | 0.429 |
| Bagging | MOE | Training set | 0.879 | 0.531 | 0.075 | 0.986 | 0.137 | 0.708 | 0.421 |
|  |  | Test set | 0.818 | 0.554 | 0.125 | 0.982 | 0.220 | 0.645 | 0.625 |
|  | ECFP6 | Training set | 0.930 | 0.568 | 0.145 | 0.991 | 0.261 | 0.632 | 0.563 |
|  |  | Test set | 0.797 | 0.520 | 0.075 | 0.965 | 0.078 | 0.609 | 0.333 |
|  | MACCS | Training set | 0.929 | 0.571 | 0.153 | 0.989 | 0.253 | 0.646 | 0.514 |
|  |  | Test set | 0.813 | 0.551 | 0.125 | 0.976 | 0.196 | 0.547 | 0.556 |
| Stratified Bagging | **MOE** | **Training set** | **0.842** | **0.800** | **0.750** | **0.849** | **0.392** | **0.814** | **0.278** |
|  |  | **Test set** | **0.813** | **0.856** | **0.925** | **0.787** | **0.588** | **0.915** | **0.507** |
|  | ECFP6 | **Training set** | **0.882** | **0.747** | **0.589** | **0.905** | **0.379** | **0.789** | **0.324** |
|  |  | Test set | 0.818 | 0.611 | 0.275 | 0.947 | 0.297 | 0.772 | 0.550 |
|  | MACCS | **Training set** | **0.798** | **0.724** | **0.637** | **0.811** | **0.278** | **0.800** | **0.207** |
|  |  | Test set | 0.789 | 0.679 | 0.500 | 0.858 | 0.345 | 0.817 | 0.455 |
| CostSensitive  Classifier | **MOE** | **10 CV** | **0.874** | **0.802** | **0.718** | **0.886** | **0.428** | **0.873** | **0.327** |
|  |  | **Test set** | **0.852** | **0.842** | **0.825** | **0.858** | **0.603** | **0.9** | **0.579** |
|  | **ECFP6** | **10 CV** | **0.647** | **0.725** | **0.815** | **0.634** | **0.237** | **0.814** | **0.147** |
|  |  | **Test set** | **0.727** | **0.698** | **0.650** | **0.746** | **0.331** | **0.766** | **0.377** |
|  | **MACCS** | **10 CV** | **0.733** | **0.737** | **0.742** | **0.732** | **0.267** | **0.819** | **0.177** |
|  |  | **Test set** | **0.761** | **0.728** | **0.675** | **0.781** | **0.389** | **0.818** | **0.422** |
| MetaCost | **MOE** | **10 CV** | **0.863** | **0.796** | **0.718** | **0.874** | **0.409** | **0.872** | **0.307** |
|  |  | **Test set** | **0.837** | **0.842** | **0.850** | **0.834** | **0.589** | **0.894** | **0.548** |
|  | **ECFP6** | **10 CV** | **0.683** | **0.736** | **0.798** | **0.674** | **0.254** | **0.796** | **0.159** |
|  |  | **Test set** | **0.670** | **0.634** | **0.575** | **0.692** | **0.219** | **0.742** | **0.307** |
|  | **MACCS** | **10 CV** | **0.717** | **0.751** | **0.790** | **0.711** | **0.277** | **0.816** | **0.175** |
|  |  | **Test set** | **0.718** | **0.721** | **0.725** | **0.716** | **0.36** | **0.767** | **0.377** |
| Threshold  Selector | **MOE** | **10 CV** | **0.908** | **0.754** | **0.573** | **0.934** | **0.433** | **0.868** | **0.403** |
|  |  | **Test set** | **0.847** | **0.791** | **0.700** | **0.882** | **0.544** | **0.912** | **0.583** |
|  | ECFP6 | **10 CV** | **0.912** | **0.722** | **0.500** | **0.944** | **0.406** | **0.838** | **0.411** |
|  |  | Test set | 0.804 | 0.583 | 0.225 | 0.941 | 0.227 | 0.795 | 0.474 |
|  | MACCS | 10 CV | 0.915 | 0.676 | 0.395 | 0.956 | 0.356 | 0.814 | 0.408 |
|  |  | Test set | 0.813 | 0.627 | 0.325 | 0.929 | 0.308 | 0.821 | 0.52 |
| SMOTE | MOE | 10 CV | 0.886 | 0.686 | 0.452 | 0.92 | 0.311 | 0.742 | 0.304 |
|  |  | **Test set** | **0.837** | **0.728** | **0.55** | **0.905** | **0.464** | **0.886** | **0.579** |
|  | ECFP6 | 10 CV | 0.926 | 0.585 | 0.185 | 0.984 | 0.263 | 0.829 | 0.469 |
|  |  | Test set | 0.804 | 0.526 | 0.075 | 0.976 | 0.112 | 0.823 | 0.429 |
|  | MACCS | 10 CV | 0.922 | 0.638 | 0.306 | 0.97 | 0.328 | 0.831 | 0.442 |
|  |  | Test set | 0.809 | 0.548 | 0.125 | 0.97 | 0.176 | 0.852 | 0.5 |
| ClassBalancer | MOE | 10 CV | 0.918 | 0.695 | 0.435 | 0.955 | 0.388 | 0.873 | 0.429 |
|  |  | Test set | 0.837 | 0.699 | 0.475 | 0.923 | 0.435 | 0.892 | 0.594 |
|  | ECFP6 | 10 CV | 0.923 | 0.643 | 0.315 | 0.971 | 0.338 | 0.827 | 0.453 |
|  |  | Test set | 0.794 | 0.539 | 0.125 | 0.953 | 0.126 | 0.815 | 0.385 |
|  | MACCS | 10 CV | 0.922 | 0.638 | 0.306 | 0.97 | 0.328 | 0.831 | 0.442 |
|  |  | Test set | 0.813 | 0.57 | 0.175 | 0.964 | 0.227 | 0.827 | 0.538 |

**Table S4.** Results on Cholestasis human dataset for all calculated statistics metrics : Accuracy, Balanced Accuracy, Sensitivity, Specificity, MCC, AUC, Precision. The performance is given for both 10-fold cross-validation and on the external test set. With bold font are depicted those models that gave a satisfactory result of sensitivity > 0.5 and they were further investigated by performing 20 iterations.

| Model Settings | Descriptors | Validation | Accuracy | Balanced Accuracy | Sensitivity | Specificity | MCC | AUC | Precision |
| --- | --- | --- | --- | --- | --- | --- | --- | --- | --- |
| Random Forest | MOE | 10 CV | 0.839 | 0.622 | 0.265 | 0.979 | 0.382 | 0.772 | 0.754 |
|  |  | **Test set** | **0.835** | **0.728** | **0.528** | **0.927** | **0.501** | **0.81** | **0.683** |
|  | ECFP6 | 10 CV | 0.833 | 0.606 | 0.231 | 0.98 | 0.35 | 0.773 | 0.741 |
|  |  | **Test set** | **0.823** | **0.719** | **0.528** | **0.91** | **0.469** | **0.835** | **0.635** |
|  | MACCS | 10 CV | 0.831 | 0.635 | 0.311 | 0.958 | 0.364 | 0.774 | 0.643 |
|  |  | **Test set** | **0.861** | **0.778** | **0.623** | **0.933** | **0.589** | **0.844** | **0.733** |
| Bagging | MOE | Training set | 0.837 | 0.617 | 0.254 | 0.980 | 0.375 | 0.691 | 0.759 |
|  |  | Test set | 0.835 | 0.723 | 0.519 | 0.927 | 0.492 | 0.73 | 0.675 |
|  | ECFP6 | Training set | 0.835 | 0.613 | 0.248 | 0.979 | 0.364 | 0.701 | 0.741 |
|  |  | Test set | 0.826 | 0.717 | 0.519 | 0.916 | 0.734 | 0.471 | 0.643 |
|  | MACCS | Training set | 0.838 | 0.634 | 0.297 | 0.970 | 0.387 | 0.685 | 0.710 |
|  |  | Test set | 0.857 | 0.764 | 0.596 | 0.933 | 0.567 | 0.763 | 0.721 |
| Stratified Bagging +cost2:1 | **MOE** | **Training set** | **0.781** | **0.719** | **0.617** | **0.821** | **0.394** | **0.768** | **0.457** |
|  |  | **Test set** | **0.761** | **0.716** | **0.635** | **0.798** | **0.395** | **0.747** | **0.478** |
|  | **ECFP6** | **Training set** | **0.804** | **0.717** | **0.573** | **0.860** | **0.413** | **0.773** | **0.501** |
|  |  | **Test set** | **0.791** | **0.736** | **0.635** | **0.837** | **0.445** | **0.761** | **0.532** |
|  | **MACCS** | **Training set** | **0.785** | **0.728** | **0.634** | **0.822** | **0.410** | **0.775** | **0.466** |
|  |  | **Test set** | **0.774** | **0.752** | **0.712** | **0.792** | **0.451** | **0.807** | **0.500** |
| CostSensitive  Classifier | **MOE** | **10 CV** | **0.724** | **0.701** | **0.663** | **0.739** | **0.337** | **0.78** | **0.383** |
|  |  | **Test set** | **0.797** | **0.769** | **0.717** | **0.82** | **0.492** | **0.795** | **0.543** |
|  | **ECFP6** | **10 CV** | **0.773** | **0.714** | **0.614** | **0.813** | **0.381** | **0.789** | **0.445** |
|  |  | **Test set** | **0.810** | **0.751** | **0.642** | **0.860** | **0.483** | **0.825** | **0.576** |
|  | **MACCS** | **10 CV** | **0.751** | **0.710** | **0.643** | **0.777** | **0.362** | **0.78** | **0.414** |
|  |  | **Test set** | **0.775** | **0.741** | **0.679** | **0.803** | **0.44** | **0.823** | **0.507** |
| MetaCost | **MOE** | **10 CV** | **0.669** | **0.670** | **0.671** | **0.668** | **0.276** | **0.741** | **0.331** |
|  |  | **Test set** | **0.697** | **0.678** | **0.642** | **0.713** | **0.310** | **0.724** | **0.400** |
|  | **ECFP6** | **10 CV** | **0.750** | **0.697** | **0.608** | **0.785** | **0.343** | **0.762** | **0.409** |
|  |  | **Test set** | **0.684** | **0.682** | **0.679** | **0.685** | **0.313** | **0.746** | **0.391** |
|  | **MACCS** | **10 CV** | **0.694** | **0.696** | **0.700** | **0.692** | **0.32** | **0.771** | **0.357** |
|  |  | **Test set** | **0.701** | **0.707** | **0.717** | **0.697** | **0.355** | **0.773** | **0.413** |
| Threshold  Selector | **MOE** | **10 CV** | **0.798** | **0.670** | **0.536** | **0.863** | **0.385** | **0.771** | **0.488** |
|  |  | **Test set** | **0.831** | **0.771** | **0.660** | **0.882** | **0.532** | **0.81** | **0.625** |
|  | ECFP6 | 10 CV | 0.816 | 0.683 | 0.464 | 0.902 | 0.387 | 0.77 | 0.537 |
|  |  | **Test set** | **0.827** | **0.762** | **0.642** | **0.882** | **0.517** | **0.835** | **0.618** |
|  | **MACCS** | **10 CV** | **0.775** | **0.702** | **0.582** | **0.822** | **0.368** | **0.774** | **0.445** |
|  |  | **Test set** | **0.805** | **0.761** | **0.679** | **0.843** | **0.490** | **0.844** | **0.563** |
| SMOTE | MOE | **10 CV** | **0.780** | **0.697** | **0.559** | **0.834** | **0.364** | **0.785** | **0.451** |
|  |  | **Test set** | **0.810** | **0.744** | **0.623** | **0.865** | **0.476** | **0.825** | **0.579** |
|  | ECFP6 | 10 CV | 0.835 | 0.748 | 0.308 | 0.965 | 0.381 | 0.777 | 0.682 |
|  |  | **Test set** | **0.836** | **0.637** | **0.585** | **0.910** | **0.517** | **0.849** | **0.660** |
|  | MACCS | 10 CV | 0.818 | 0.651 | 0.375 | 0.927 | 0.353 | 0.774 | 0.556 |
|  |  | **Test set** | **0.848** | **0.776** | **0.642** | **0.91** | **0.563** | **0.849** | **0.68** |
| ClassBalancer | MOE | 10 CV | 0.824 | 0.697 | 0.438 | 0.919 | 0.396 | 0.776 | 0.569 |
|  |  | Test set | 0.844 | 0.773 | 0.642 | 0.904 | 0.554 | 0.788 | 0.667 |
|  | ECFP6 | 10 CV | 0.840 | 0.687 | 0.435 | 0.939 | 0.437 | 0.78 | 0.637 |
|  |  | **Test set** | **0.827** | **0.749** | **0.604** | **0.893** | **0.504** | **0.833** | **0.627** |
|  | MACCS | 10 CV | 0.809 | 0.678 | 0.464 | 0.893 | 0.371 | 0.776 | 0.514 |
|  |  | **Test set** | **0.835** | **0.774** | **0.660** | **0.888** | **0.541** | **0.835** | **0.636** |

**Table S5.** Results on Cholestasis animal dataset for all calculated statistics metrics : Accuracy, Balanced Accuracy, Sensitivity, Specificity, MCC, AUC, Precision. The performance is given for both 10-fold cross-validation and on the external test set. With bold font are depicted those models that gave a satisfactory result of sensitivity > 0.5 and they were further investigated by performing 20 iterations.

| Model Settings | Descriptors | Validation | Accuracy | Balanced Accuracy | Sensitivity | Specificity | MCC | AUC | Precision |
| --- | --- | --- | --- | --- | --- | --- | --- | --- | --- |
| Random Forest | MOE | 10 CV | 0.953 | 0.500 | 0.000 | 1.000 | 0.000 | 0.703 | 0.000 |
|  | ECFP6 | 10 CV | 0.953 | 0.500 | 0.000 | 1.000 | 0.000 | 0.629 | 0.000 |
|  | MACCS | 10 CV | 0.951 | 0.511 | 0.027 | 0.997 | 0.083 | 0.700 | 0.333 |
| Bagging | MOE | Training set | 0.952 | 0.500 | 0.000 | 0.999 | -0.006 | 0.503 | 0.000 |
|  | ECFP6 | Training set | 0.953 | 0.500 | 0.000 | 1.000 | 0.000 | 0.498 | 0.000 |
|  | MACCS | Training set | 0.952 | 0.512 | 0.027 | 0.998 | 0.093 | 0.521 | 0.400 |
| Stratified Bagging +cost2:1 | **MOE** | **Training set** | **0.636** | **0.594** | **0.547** | **0.641** | **0.083** | **0.715** | **0.070** |
|  | **ECFP6** | **Training set** | **0.722** | **0.639** | **0.547** | **0.731** | **0.131** | **0.686** | **0.092** |
|  | **MACCS** | **Training set** | **0.623** | **0.637** | **0.653** | **0.621** | **0.119** | **0.732** | **0.079** |
| CostSensitive  Classifier | **MOE** | **10 CV** | **0.632** | **0.623** | **0.613** | **0.633** | **0.108** | **0.665** | **0.077** |
|  | **ECFP6** | **10 CV** | **0.532** | **0.527** | **0.520** | **0.533** | **0.023** | **0.531** | **0.052** |
|  | **MACCS** | **10 CV** | **0.579** | **0.633** | **0.693** | **0.573** | **0.114** | **0.690** | **0.075** |
| MetaCost | **MOE** | **10 CV** | **0.582** | **0.597** | **0.613** | **0.580** | **0.083** | **0.644** | **0.068** |
|  | **ECFP6** | **10 CV** | **0.599** | **0.587** | **0.573** | **0.600** | **0.075** | **0.600** | **0.066** |
|  | **MACCS** | **10 CV** | **0.588** | **0.645** | **0.707** | **0.582** | **0.124** | **0.674** | **0.077** |
| Threshold  Selector | MOE | 10 CV | 0.875 | 0.580 | 0.253 | 0.906 | 0.112 | 0.686 | 0.118 |
|  | ECFP6 | 10 CV | 0.874 | 0.567 | 0.227 | 0.906 | 0.094 | 0.624 | 0.107 |
|  | MACCS | 10 CV | 0.848 | 0.635 | 0.4 | 0.87 | 0.163 | 0.687 | 0.132 |
| SMOTE | MOE | 10 CV | 0.943 | 0.533 | 0.080 | 0.985 | 0.105 | 0.728 | 0.214 |
|  | ECFP6 | 10 CV | 0.953 | 0.500 | 0.000 | 1.000 | 0.000 | 0.638 | 0.000 |
|  | MACCS | 10 CV | 0.949 | 0.511 | 0.027 | 0.995 | 0.057 | 0.708 | 0.2 |
| ClassBalancer | MOE | 10 CV | 0.948 | 0.697 | 0.4 | 0.993 | 0.079 | 0.681 | 0.231 |
|  | ECFP6 | 10 CV | 0.951 | 0.505 | 0.013 | 0.997 | 0.04 | 0.636 | 0.2 |
|  | MACCS | 10 CV | 0.945 | 0.509 | 0.027 | 0.991 | 0.037 | 0.693 | 0.125 |

**Table S6.** Results on OATP1B1 inhibition dataset only for the best performing methods on the appropriate set of descriptors (Sensitivity ≥ 0.5) for all calculated statistics metrics : Accuracy, Balanced Accuracy, Sensitivity, Specificity, MCC, AUC, Precision. The mean performance out of 20 iterations and the standard deviation values are provided.

| Model Settings | Descriptors | Statistical Value | Accuracy | Balanced Accuracy | Sensitivity | Specificity | MCC | AUC | Precision |
| --- | --- | --- | --- | --- | --- | --- | --- | --- | --- |
| -Stratified Bagging | MOE | mean | 0.769 | 0.817 | 0.823 | 0.810 | 0.334 | 0.715 | 0.334 |
|  |  | sd | 0.002 | 0.005 | 0.010 | 0.002 | 0.007 | 0.005 | 0.004 |
|  | ECFP6 | mean | 0.805 | 0.734 | 0.642 | 0.826 | 0.351 | 0.795 | 0.315 |
|  |  | sd | 0.002 | 0.005 | 0.009 | 0.003 | 0.007 | 0.006 | 0.004 |
|  | MACCS | mean | 0.721 | 0.724 | 0.728 | 0.721 | 0.299 | 0.803 | 0.245 |
|  |  | sd | 0.003 | 0.004 | 0.007 | 0.003 | 0.006 | 0.004 | 0.003 |
| CostSensitive  Classifier | MOE | mean | 0.847 | 0.754 | 0.634 | 0.873 | 0.413 | 0.804 | 0.385 |
|  |  | sd | 0.003 | 0.011 | 0.020 | 0.003 | 0.016 | 0.067 | 0.008 |
|  | ECFP6 | mean | 0.641 | 0.701 | 0.778 | 0.624 | 0.256 | 0.785 | 0.206 |
|  |  | sd | 0.009 | 0.014 | 0.018 | 0.010 | 0.013 | 0.007 | 0.006 |
|  | MACCS | mean | 0.646 | 0.707 | 0.784 | 0.629 | 0.264 | 0.798 | 0.212 |
|  |  | sd | 0.005 | 0.011 | 0.017 | 0.005 | 0.011 | 0.006 | 0.013 |
| MetaCost | MOE | mean | 0.817 | 0.747 | 0.656 | 0.837 | 0.376 | 0.822 | 0.335 |
|  |  | sd | 0.006 | 0.009 | 0.011 | 0.006 | 0.011 | 0.005 | 0.009 |
|  | ECFP6 | mean | 0.625 | 0.694 | 0.782 | 0.605 | 0.245 | 0.770 | 0.201 |
|  |  | sd | 0.008 | 0.013 | 0.017 | 0.008 | 0.012 | 0.005 | 0.012 |
|  | MACCS | mean | 0.666 | 0.705 | 0.755 | 0.655 | 0.264 | 0.772 | 0.215 |
|  |  | sd | 0.007 | 0.011 | 0.014 | 0.008 | 0.011 | 0.005 | 0.005 |
| Threshold  Selector | MOE | mean | 0.879 | 0.721 | 0.519 | 0.924 | 0.420 | 0.813 | 0.460 |
|  |  | sd | 0.005 | 0.017 | 0.027 | 0.006 | 0.018 | 0.007 | 0.018 |
|  | ECFP6 | mean | 0.875 | 0.703 | 0.483 | 0.924 | 0.391 | 0.794 | 0.442 |
|  |  | sd | 0.005 | 0.014 | 0.021 | 0.007 | 0.015 | 0.007 | 0.017 |
| SMOTE | MOE | mean | 0.870 | 0.715 | 0.517 | 0.914 | 0.398 | 0.811 | 0.430 |
|  |  | sd | 0.005 | 0.011 | 0.017 | 0.005 | 0.019 | 0.003 | 0.018 |

**Table S7.** Results on OATP1B3 inhibition dataset only for the best performing methods on the appropriate set of descriptors (Sensitivity ≥ 0.5) for all calculated statistics metrics : Accuracy, Balanced Accuracy, Sensitivity, Specificity, MCC, AUC, Precision. The mean performance out of 20 iterations and the standard deviation values are provided.

| Model Settings | Descriptors | Statistical Value | Accuracy | Balanced Accuracy | Sensitivity | Specificity | MCC | AUC | Precision |
| --- | --- | --- | --- | --- | --- | --- | --- | --- | --- |
| Stratified Bagging | MOE | mean | 0.841 | 0.804 | 0.761 | 0.847 | 0.395 | 0.819 | 0.278 |
|  |  | sd | 0.002 | 0.005 | 0.044 | 0.004 | 0.060 | 0.031 | 0.004 |
|  | ECFP6 | mean | 0.882 | 0.755 | 0.606 | 0.904 | 0.388 | 0.789 | 0.328 |
|  |  | sd | 0.002 | 0.005 | 0.010 | 0.002 | 0.008 | 0.009 | 0.006 |
|  | MACCS | mean | 0.799 | 0.729 | 0.647 | 0.811 | 0.285 | 0.800 | 0.210 |
|  |  | sd | 0.003 | 0.010 | 0.019 | 0.003 | 0.012 | 0.008 | 0.006 |
| CostSensitive  Classifier | MOE | mean | 0.871 | 0.792 | 0.695 | 0.890 | 0.420 | 0.874 | 0.328 |
|  |  | sd | 0.024 | 0.009 | 0.015 | 0.003 | 0.010 | 0.0037 | 0.007 |
|  | ECFP6 | mean | 0.651 | 0.725 | 0.811 | 0.639 | 0.238 | 0.809 | 0.148 |
|  |  | sd | 0.009 | 0.014 | 0.018 | 0.010 | 0.010 | 0.008 | 0.004 |
|  | MACCS | mean | 0.737 | 0.729 | 0.720 | 0.739 | 0.260 | 0.820 | 0.176 |
|  |  | sd | 0.006 | 0.013 | 0.021 | 0.006 | 0.013 | 0.007 | 0.005 |
| MetaCost | MOE | mean | 0.864 | 0.7964 | 0.7174 | 0.8754 | 0.410 | 0.870 | 0.308 |
|  |  | sd | 0.003 | 0.012 | 0.021 | 0.003 | 0.013 | 0.004 | 0.008 |
|  | ECFP6 | mean | 0.688 | 0.732 | 0.783 | 0.681 | 0.251 | 0.797 | 0.160 |
|  |  | sd | 0.007 | 0.012 | 0.019 | 0.006 | 0.012 | 0.005 | 0.004 |
|  | MACCS | mean | 0.711 | 0.738 | 0.769 | 0.706 | 0.262 | 0.802 | 0.169 |
|  |  | sd | 0.007 | 0.016 | 0.024 | 0.007 | 0.014 | 0.009 | 0.006 |
| Threshold  Selector | MOE | mean | 0.907 | 0.748 | 0.562 | 0.934 | 0.423 | 0.872 | 0.397 |
|  |  | sd | 0.004 | 0.024 | 0.041 | 0.006 | 0.022 | 0.006 | 0.017 |
|  | ECFP6 | mean | 0.903 | 0.725 | 0.518 | 0.937 | 0.388 | 0.820 | 0.374 |
|  |  | sd | 0.006 | 0.018 | 0.030 | 0.007 | 0.022 | 0.012 | 0.021 |

**Table S8.** Results on human cholestasis dataset only for the best performing methods on the appropriate set of descriptors (Sensitivity ≥ 0.5) for all calculated statistics metrics : Accuracy, Balanced Accuracy, Sensitivity, Specificity, MCC, AUC, Precision. The mean performance out of 20 iterations and the standard deviation values are provided.

| Model Settings | Descriptors | Statistical Value | Accuracy | Balanced Accuracy | Sensitivity | Specificity | MCC | AUC | Precision |
| --- | --- | --- | --- | --- | --- | --- | --- | --- | --- |
| Stratified Bagging +cost2:1 | MOE | mean | 0.777 | 0.713 | 0.607 | 0.819 | 0.384 | 0.768 | 0.450 |
|  |  | sd | 0.002 | 0.005 | 0.011 | 0.003 | 0.008 | 0.004 | 0.005 |
|  | ECFP6 | mean | 0.806 | 0.722 | 0.583 | 0.860 | 0.421 | 0.773 | 0.505 |
|  |  | sd | 0.003 | 0.003 | 0.006 | 0.004 | 0.006 | 0.004 | 0.006 |
|  | MACCS | mean | 0.782 | 0.723 | 0.625 | 0.820 | 0.400 | 0.772 | 0.460 |
|  |  | sd | 0.005 | 0.004 | 0.007 | 0.005 | 0.009 | 0.005 | 0.008 |
| CostSensitive  Classifier | MOE | mean | 0.731 | 0.707 | 0.667 | 0.74685 | 0.346 | 0.786 | 0.392 |
|  |  | sd | 0.005 | 0.009 | 0.013 | 0.005 | 0.011 | 0.005 | 0.006 |
|  | ECFP6 | mean | 0.771 | 0.704 | 0.596 | 0.812 | 0.369 | 0.782 | 0.440 |
|  |  | sd | 0.006 | 0.014 | 0.017 | 0.012 | 0.015 | 0.006 | 0.011 |
|  | MACCS | mean | 0.753 | 0.705 | 0.629 | 0.782 | 0.343 | 0.776 | 0.415 |
|  |  | sd | 0.006 | 0.010 | 0.011 | 0.010 | 0.073 | 0.005 | 0.008 |
| MetaCost | MOE | mean | 0.671 | 0.681 | 0.698 | 0.664 | 0.293 | 0.755 | 0.337 |
|  |  | sd | 0.008 | 0.014 | 0.019 | 0.010 | 0.015 | 0.007 | 0.007 |
|  | ECFP6 | mean | 0.750 | 0.699 | 0.614 | 0.783 | 0.346 | 0.768 | 0.409 |
|  |  | sd | 0.006 | 0.011 | 0.015 | 0.007 | 0.013 | 0.006 | 0.009 |
|  | MACCS | mean | 0.690 | 0.692 | 0.695 | 0.689 | 0.313 | 0.764 | 0.353 |
|  |  | sd | 0.005 | 0.006 | 0.013 | 0.007 | 0.010 | 0.004 | 0.006 |
| Threshold  Selector | MOE | mean | 0.801 | 0.694 | 0.518 | 0.870 | 0.382 | 0.775 | 0.495 |
|  |  | sd | 0.008 | 0.013 | 0.015 | 0.011 | 0.015 | 0.006 | 0.018 |
|  | MACCS | mean | 0.782 | 0.694 | 0.551 | 0.838 | 0.363 | 0.771 | 0.455 |
|  |  | sd | 0.008 | 0.021 | 0.027 | 0.015 | 0.011 | 0.005 | 0.014 |

**Table S9.** Results animal cholestasis dataset only for the best performing methods on the appropriate set of descriptors (Sensitivity ≥ 0.5) for all calculated statistics metrics : Accuracy, Balanced Accuracy, Sensitivity, Specificity, MCC, AUC, Precision. The mean performance out of 20 iterations and the standard deviation values are provided.

| Model Settings | Descriptors | Statistical Value | Accuracy | Balanced Accuracy | Sensitivity | Specificity | MCC | AUC | Precision |
| --- | --- | --- | --- | --- | --- | --- | --- | --- | --- |
| Stratified Bagging +cost2:1 | MOE | mean | 0.648 | 0.608 | 0.564 | 0.653 | 0.096 | 0.710 | 0.075 |
|  |  | sd | 0.015 | 0.011 | 0.021 | 0.016 | 0.010 | 0.008 | 0.003 |
|  | ECFP6 | mean | 0.713 | 0.633 | 0.545 | 0.721 | 0.124 | 0.678 | 0.088 |
|  |  | sd | 0.009 | 0.008 | 0.018 | 0.010 | 0.008 | 0.009 | 0.003 |
|  | MACCS | mean | 0.624 | 0.636 | 0.649 | 0.623 | 0.118 | 0.729 | 0.079 |
|  |  | sd | 0.007 | 0.009 | 0.022 | 0.008 | 0.008 | 0.008 | 0.002 |
| CostSensitive  Classifier | MOE | mean | 0.6304 | 0.6122 | 0.592 | 0.632 | 0.098 | 0.659 | 0.074 |
|  |  | sd | 0.009 | 0.017 | 0.030 | 0.009 | 0.015 | 0.015 | 0.005 |
|  | ECFP6 | mean | 0.530 | 0.533 | 0.536 | 0.523 | 0.026 | 0.541 | 0.053 |
|  |  | sd | 0.008 | 0.023 | 0.048 | 0.008 | 0.023 | 0.014 | 0.004 |
|  | MACCS | mean | 0.588 | 0.645 | 0.708 | 0.582 | 0.125 | 0.683 | 0.078 |
|  |  | sd | 0.008 | 0.022 | 0.044 | 0.008 | 0.019 | 0.018 | 0.005 |
| MetaCost | MOE | mean | 0.586 | 0.610 | 0.637 | 0.5829 | 0.095 | 0.666 | 0.070 |
|  |  | sd | 0.009 | 0.014 | 0.018 | 0.009 | 0.009 | 0.011 | 0.003 |
|  | ECFP6 | mean | 0.587 | 0.599 | 0.6126 | 0.586 | 0.085 | 0.610 | 0.098 |
|  |  | sd | 0.014 | 0.032 | 0.048 | 0.016 | 0.019 | 0.025 | 0.005 |
|  | MACCS | mean | 0.5894 | 0.6453 | 0.708 | 0.5826 | 0.1245 | 0.6752 | 0.0776 |
|  |  | sd | 0.008 | 0.024 | 0.039 | 0.010 | 0.016 | 0.012 | 0.004 |

**Figures**

**Figure S1 (a-d).** Comparison of performances of different meta-classifiers on the four training datasets (after one round of 10-fold cross validation). *x-axis* corresponds to the sensitivity and on the *y-axis* is the specificity. The squares correspond to MOE descriptors, the triangles correspond to ECFP6 fingerprints and the circles correspond to MACCS fingerprints. Each classifier is depicted in a different color: red for RF standalone, green for Bagging, blue for Stratified Bagging, dark pink for CostSensitiveClassifier, cyan for MetaCost, yellow for ThresholdSelector, orange for SMOTE and dark violet for ClassBalancer.

(a)
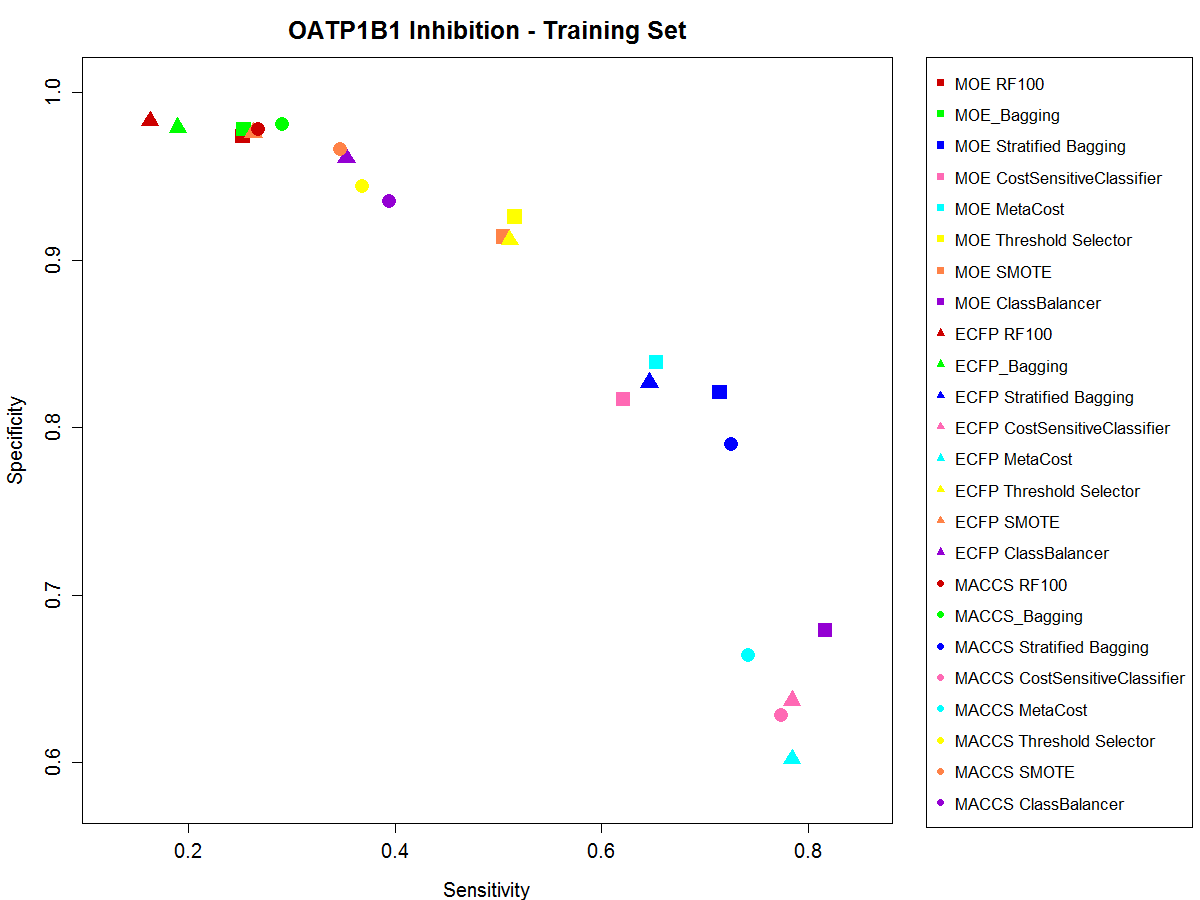
 (b)
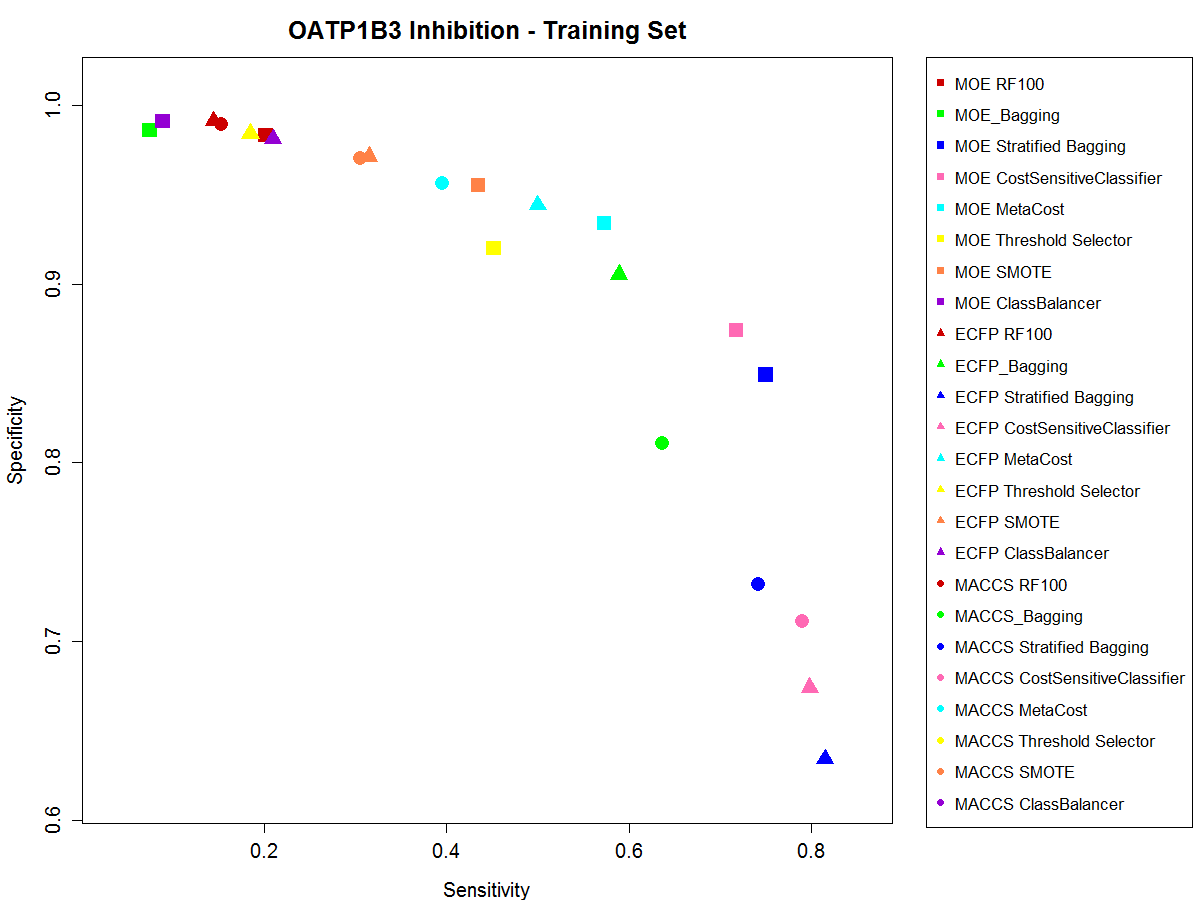


(c)
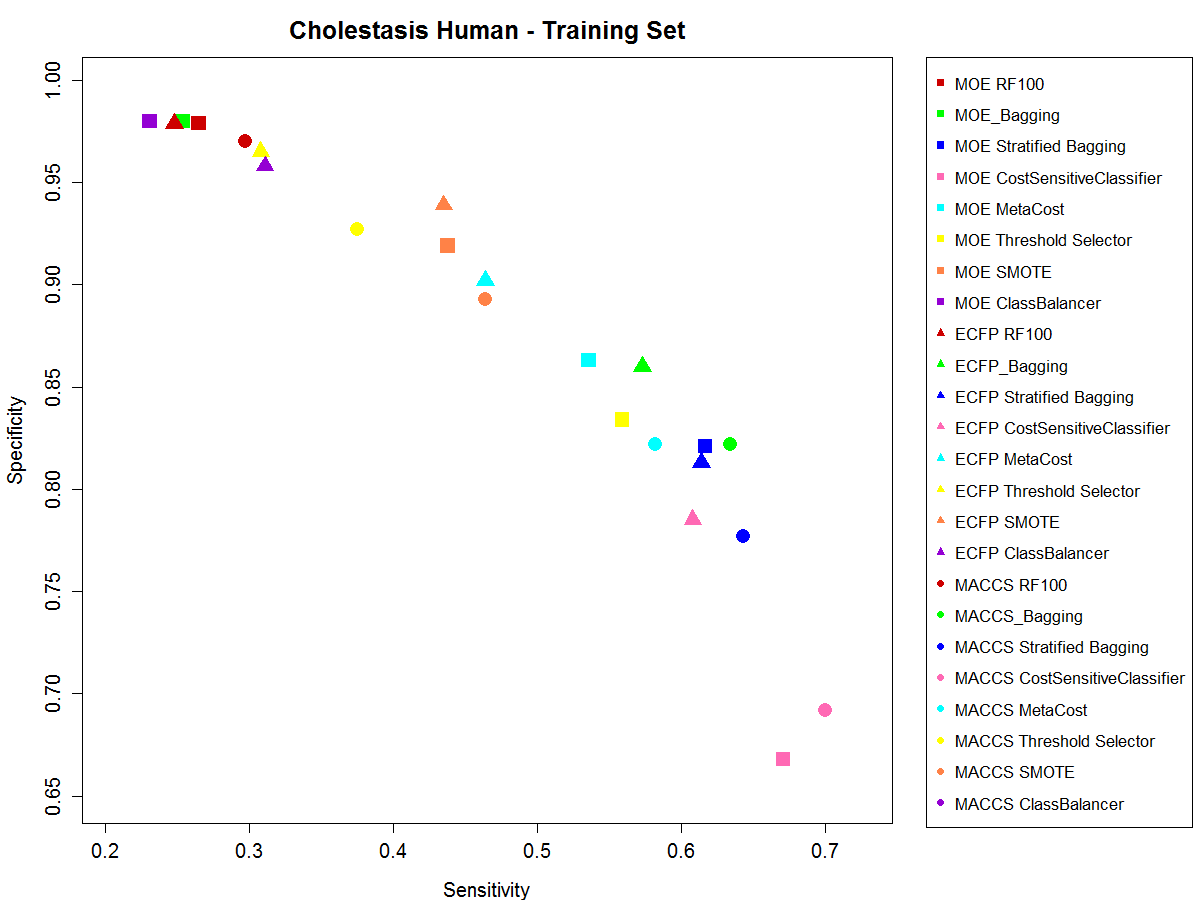
 (d)
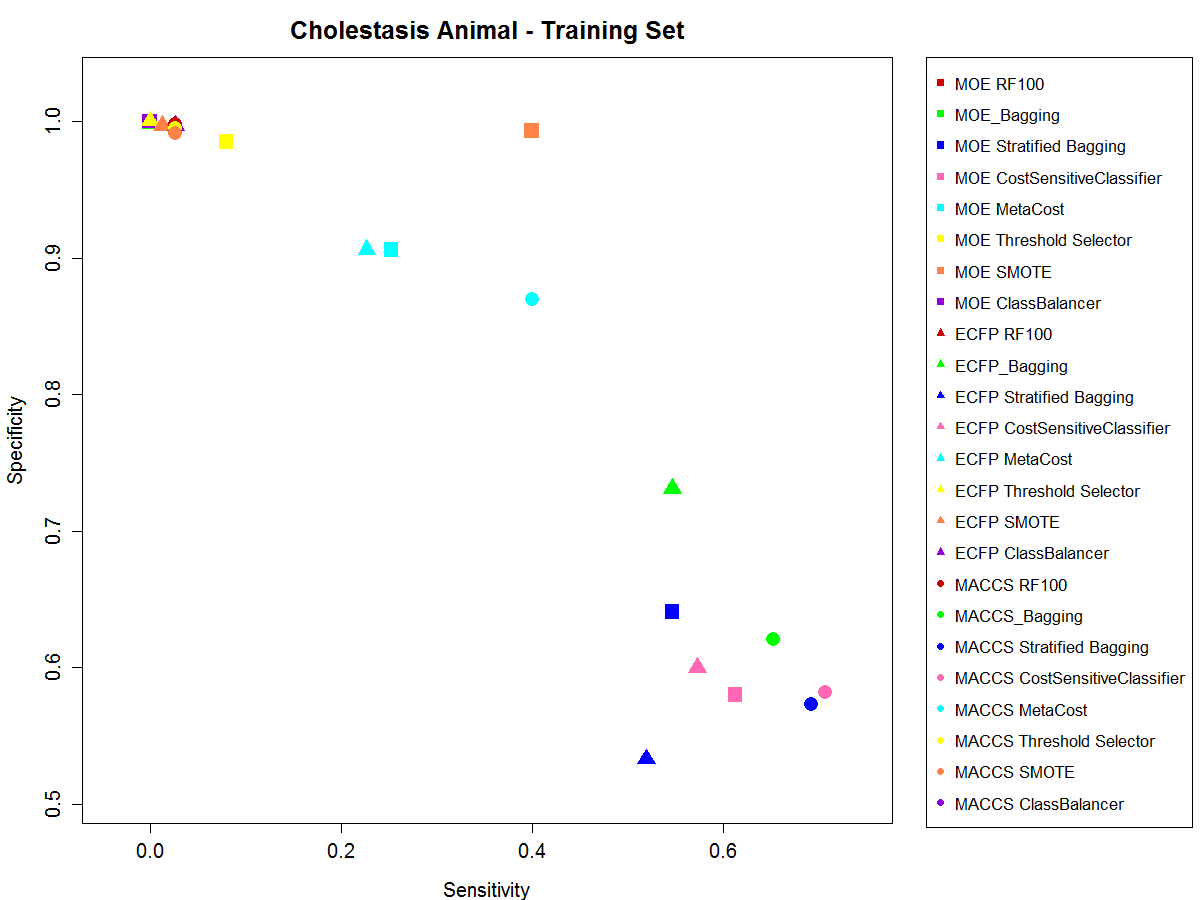

Supplement: Supplementary file 1 — Supplementary material 1 (DOCX 167 KB) [file 10822_2018_116_MOESM1_ESM.docx]
